# *Staphylococcus aureus* persistence in the lung is necessary to elicit systemic protective immunity

**DOI:** 10.3389/fimmu.2025.1587018

**Published:** 2025-09-18

**Authors:** Usha Vyshnavi Chidella, Zhaotao Li, Maureen Kleinhenz, Christopher P. Montgomery

**Affiliations:** ^1^ Center for Microbe and Immunity Research, Abigail Wexner Research Institute at Nationwide Children’s Hospital, Columbus, OH, United States; ^2^ Department of Pediatrics, College of Medicine, The Ohio State University, Columbus, OH, United States; ^3^ Division of Critical Care Medicine, Nationwide Children’s Hospital, Columbus, OH, United States

**Keywords:** *Staphylococcus aureus*, SSTI, pneumonia, antigen persistence, T cells, antibody, protective immunity

## Abstract

*Staphylococcus aureus* is a leading cause of skin and soft tissue infections (SSTI) and pneumonia. Recurrence is common and treatment is complicated by antimicrobial resistance; therefore, it is necessary to understand the mechanisms by which the host develops protective immunity against *S. aureus*. We previously reported that SSTI, but not pneumonia, elicits strong *S. aureus*-specific antibody and T cell responses and protection against recurrent infection; these findings suggested that site-specific elicited immune responses drive protective immunity. Because *S. aureus* is rapidly cleared from the lung but persists in the skin, we hypothesized that bacterial persistence in the lung is necessary to elicit protective antibody and T cell responses. In this study, we tested this hypothesis using a newly described mouse model of persistent pneumonia. Indeed, persistent pneumonia and SSTI elicited strong toxin-specific antibody and CD4^+^ IL-17^+^ and IFNγ^+^ T cell responses, whereas transient pneumonia did not. Persistence of *S. aureus* in the lung was accompanied by durable systemic T and B cell expansion observed as early as 9 days after infection. Consistent with important roles for antibodies and T cells in protective immunity, SSTI and persistent pneumonia, but not transient pneumonia, elicited protection against secondary SSTI and pneumonia. Taken together, these results demonstrate that bacterial persistence in infected tissues is necessary to elicit protective immunity against recurrent infections. These findings have important implications in better understanding the mechanisms of natural immunity against *S. aureus*.

## Introduction

*Staphylococcus aureus* is a common cause of community and health care-associated infections ranging from mild, such as skin and soft tissue infections (SSTI), to life threatening conditions including pneumonia and sepsis (1). *S. aureus* is a leading bacterial cause of death worldwide (2). In the United States, nearly 50% of patients with a *S. aureus* SSTI experience a recurrence within a year (3). While still common, recurrence is less frequent following invasive infections such as pneumonia (4). These findings suggest that the site and severity of primary infection are important determinants of elicited protective immunity and subsequent risk of recurrence.

In contrast to humans, we previously reported that non-invasive *S. aureus* SSTI elicits stronger adaptive immune responses than pneumonia (5). Specifically, whereas SSTI elicits strong antibody and T cell responses and protection against secondary infection, pneumonia fails to protect mice against secondary infection due to poorly eliciting antibodies and T cells (5). While site-specificity of the elicited immune response may play a role in differential protection, an alternative, but not mutually exclusive, explanation for these findings is that rapid clearance of bacteria from the lung following pneumonia precludes generation of a memory response. In support of this notion, persistence of *Pseudomonas aeruginosa*, *Salmonella typhimurium*, and *Chlamydia trachomitis* is necessary to generate antigen specific memory T cells (6–8). Based on these studies and our previous work, we hypothesized that bacterial persistence in the lung is necessary to elicit protective immunity against secondary *S. aureus* infection. To test this hypothesis, we developed a model of persistent *S. aureus* pneumonia. Using this model, we found that bacterial persistence in the lung was sufficient to elicit toxin-specific antibodies and T cells and protect against secondary SSTI and pneumonia.

## Materials and methods

### Mouse models of *S. aureus* infection

Our models of *S. aureus* SSTI and pneumonia have been described previously (9). BALB/c mice used in this study were purchased from Jackson labs. The virulence of the USA300 clinical isolate 923 in mouse models has been reported (8, 9). Bacterial isolates were thawed from frozen stocks, revived on tryptic soy agar (TSA) overnight at 37°C, and sub-cultured into tryptic soy broth (TSB) and grown at 37°C overnight with shaking at 250 rpm. After 12 hours, the culture was diluted 1:100 in fresh TSB and cultured for 3 hours (OD_600_ of 1.8). The culture was centrifuged at 1300g for 5 min and washed in phosphate-buffered saline (PBS), followed by resuspension in fresh PBS to obtain the desired inoculum.

One day prior to SSTI, mice were sedated by intraperitoneal injection of ketamine (17.5 mg/kg) & xylazine (2.5mg/kg) and the back was shaved and depilated. On the day of inoculation, mice received subcutaneous injection of 50μl *S. aureus* (1.5–2 x10^7^ CFU) on the left flank. For pneumonia, mice received 1 x 10^8^ CFU intranasally in 20μl. Mice in the “1x pna” groups received a single inoculum and mice in the “4x pna” groups were repeatedly inoculated every 3 days (4 total inocula). To quantify bacterial persistence in these models, groups of mice were euthanized every 3 days from day 3 to 28 after infection. Following euthanasia, whole lungs were isolated and homogenized in 1ml of pbs and blood was collected via submandibular puncture. Serial dilutions of lung homogenates and blood were plated on mannitol salt agar (MSA), incubated at 37°C for 24 hr, following which colonies were enumerated.

Secondary infections were performed 6 weeks after the primary infection. For secondary SSTI, mice were inoculated on the right flank and lesion size was quantified by digital photography daily for 7 days using isoflurane sedation(O_2_: 1L/min, 2-3% isoflurane vaporizer for 3–4 min). On day 7, mice were sacrificed, lesions were aseptically removed and homogenized in 1ml PBS, and serial dilutions plated on MSA for colony enumeration. For secondary pneumonia, mice were inoculated with 3 x 10^8^ CFU *S. aureus*. Mice were monitored daily (at least twice/day) and the severity of illness was quantified using a validated illness severity score that reliably predicts mortality (5). Moribund animals were euthanized immediately by forced CO_2_ inhalation (3.8L/min, in 38L chamber for 5–6 min).

### Quantification of antibody levels

Blood was obtained from by submandibular collection 3, 6, 9, 12, and 28 days following primary infection and serum was isolated by centrifugation at 2500g for 10min. ELISA was used to quantify antibody levels as previously reported (10). 96 well plates were coated with purified α-hemolysin (Hla; 5 ug), followed by incubation with 1:200 dilution of mouse serum and alkaline phosphatase (AP)-conjugated goat anti-mouse IgG (1:3000; Jackson ImmunoResearch). Absorbance was measured at 405 nm using a GENios spectrophotometer (Tecan).

### Quantification of T cell responses by ELISpot

As reported (11), single cell splenocyte suspensions were prepared using collagenase and DNase I. Splenocytes were seeded on ELISpot plates at concentration of 8x10^5^/well for plates coated with anti-IL-17 antibody and 4x10^5^ cells/well for anti-IFNγ antibody (BD biosciences). Splenocytes were incubated for 40 hours at 37°C with heat-killed *S. aureus* (HKSA; 5x10^5^ CFU/ml) or purified Hla, leukotoxin E (LukE), or Panton-Valentine leukocidin S (LukS-PV)(20μg/ml). For detection antibodies, Biotinylated anti-IL-17A and anti-IFNγ (BD biosciences) antibodies and HRP-Avidin (eBioscience) at 1:500 dilution was used. AEC substrate kit was used to develop spots and reaction was stopped by washing plates with DI water. Spots were analyzed by ImmunoSpot series 1 analyzer from Cellular Technology.

### Quantification of T cells by flow cytometry

*S. aureus*- and antigen-specific T cells were quantified by flow cytometry. As described above, single cell splenocyte suspensions were prepared, and cells were incubated with HKSA (2.5ul/ml of 4x10^8^ cfu), and other purified antigens (Hla, LukE, LuKS-PV) each at 2ug/ml for 40 hours. To eliminate dead cells, all cells were stained with Ghost UV-450 or LD stain (APCcy7) (Life Technologies and Biolegend, respectively). CD3 (17A2) and CD4 (GK1.5), CD19, CD44, and CXCR5 antibodies (Biolegend) were used for surface staining. CD4^+^ T cells, follicular T helper cells (CD4^+^ CXCR5^+^), activated T cells (CD3^+^ CD44^+^), and B cells (CD3^-^ CD19^+^) were quantified by flow cytometry. Cells were fixed for 30 min at RT and permeabilized with true nuclear transcription factor kit (Biolegend). For intracellular staining, cells were incubated on ice for 30 min with IL-17A (TC11-18H10) and IFNγ (XM G1.2) antibodies. Th1 cells were identified as CD4^+^ IFNγ^+^ and Th17 cell as CD4^+^ IL-17A^+^. Flow cytometry was performed on an LSRII (BD Biosciences) cytometer and data were analyzed using FlowJo. To account for differences in spleen size based on the experimental group, cell numbers were normalized to the total number of splenocytes isolated per spleen.

### Data analysis

Data were compared using one-way ANOVA with Tukey’s post-test, two-way ANOVA with repeated measures, or Gehan-Breslow-Wilcoxon test where appropriate. Differences were considered significant when *p* values were <0.05. All data analysis was performed using GraphPad Prism.

### Study approval

All animal experiments were approved by the Institutional Animal Care and Use Committee at the Abigail Wexner Research Institute at Nationwide Children’s Hospital.

## Results

### Model of *S. aureus* persistent pneumonia

**Figure 1:**
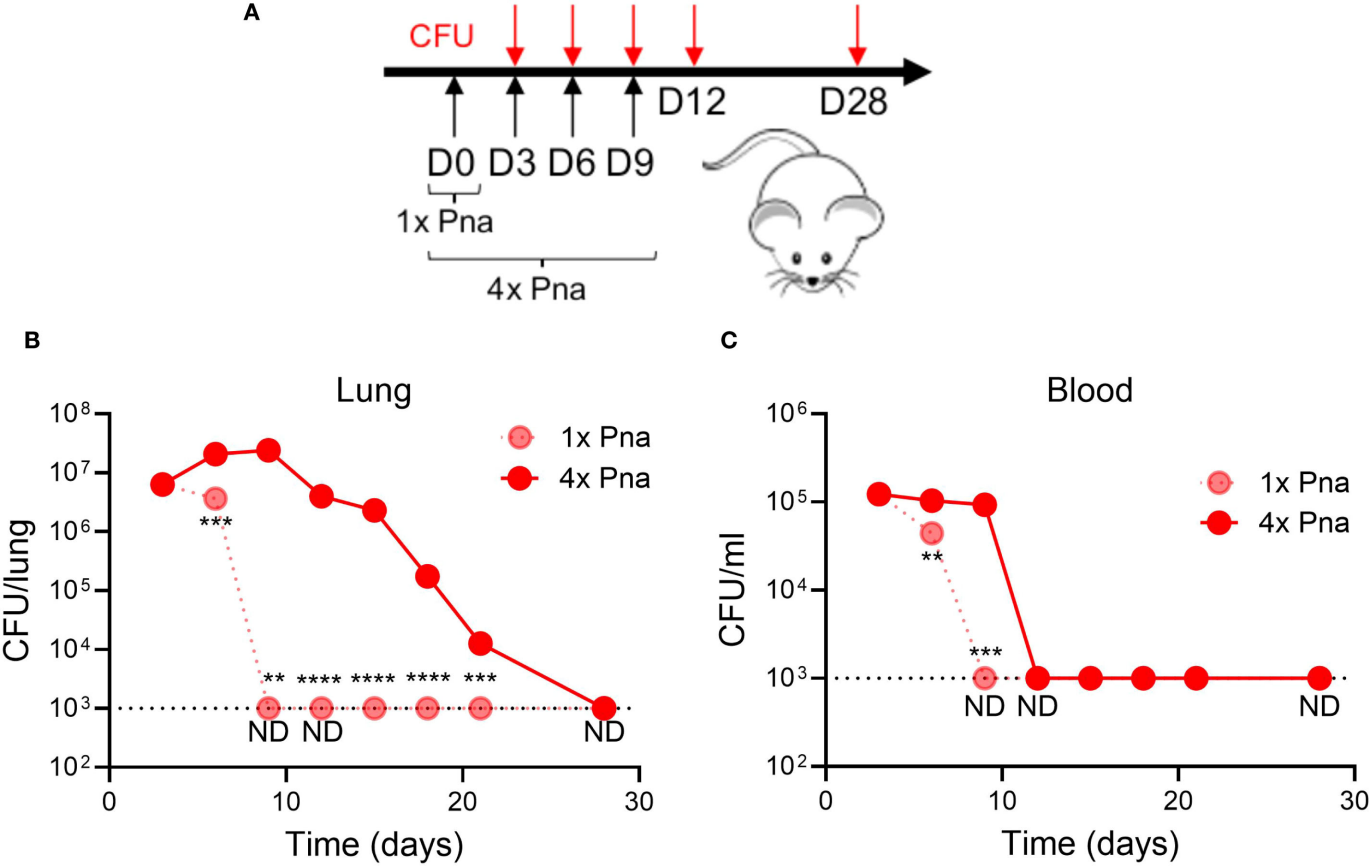
Model of persistent bacterial pneumonia. **(A)** Experimental models of transient (1x) and persistent (4x) pneumonia, for which mice received repeated sublethal inocula (1 x 10^8^ CFU) on days 3, 6, and 9. **(B)** Bacterial burden in the lung following 1x pna and 4x pna. **(C)** Bacterial burden in the blood following 1x pna and 4x pna. N=5 mice/group. Data are presented as mean ± SEM and were compared using unpaired T- test. **indicates *p*<0.01, ***p<0.001, ****p<0.0001. ND indicates not detected; the dotted line indicates the lower limit of detection.

We previously reported that, in contrast to SSTI, primary *S. aureus* pneumonia failed to elicit protective antibody and T cell responses against secondary SSTI or pneumonia (5). While tissue specificity may contribute to the differences in observed protection, we hypothesized that one explanation for the failure of pneumonia to elicit strong antibody and T cell responses is the early clearance of *S. aureus* from the lungs of infected mice. To test this hypothesis, we developed a mouse model of *S. aureus* persistence in the lung by repeated sublethal (1 x 10^8^ CFU in 20μl) intranasal inoculation every 3 days for 4 doses (**Figure 1A**). This infection regimen did not cause clinical illness in the mice, with only slight ruffled fur observed in the first 24 hours that resolved thereafter. Using this model, we found that bacterial numbers in the lung were decreased during transient pneumonia (“1x pna”) by 6 days after infection and cleared by 9 days (**Figure 1B**). In contrast, *S. aureus* persisted in the lung for at least 21 days after infection in the model of persistent pneumonia (“4x pna”) and cleared by 28 days (**Figure 1B**). Similarly, *S. aureus* was cleared from the blood before day 9 in the 1x pna group and before day 12 in the 4x pna group (**Figure 1C**). Therefore, the 4x pna model resulted in *S. aureus* persistence in the lung.

### *S. aureus* persistence in the lung elicits Hla-specific antibodies

**Figure 2:**
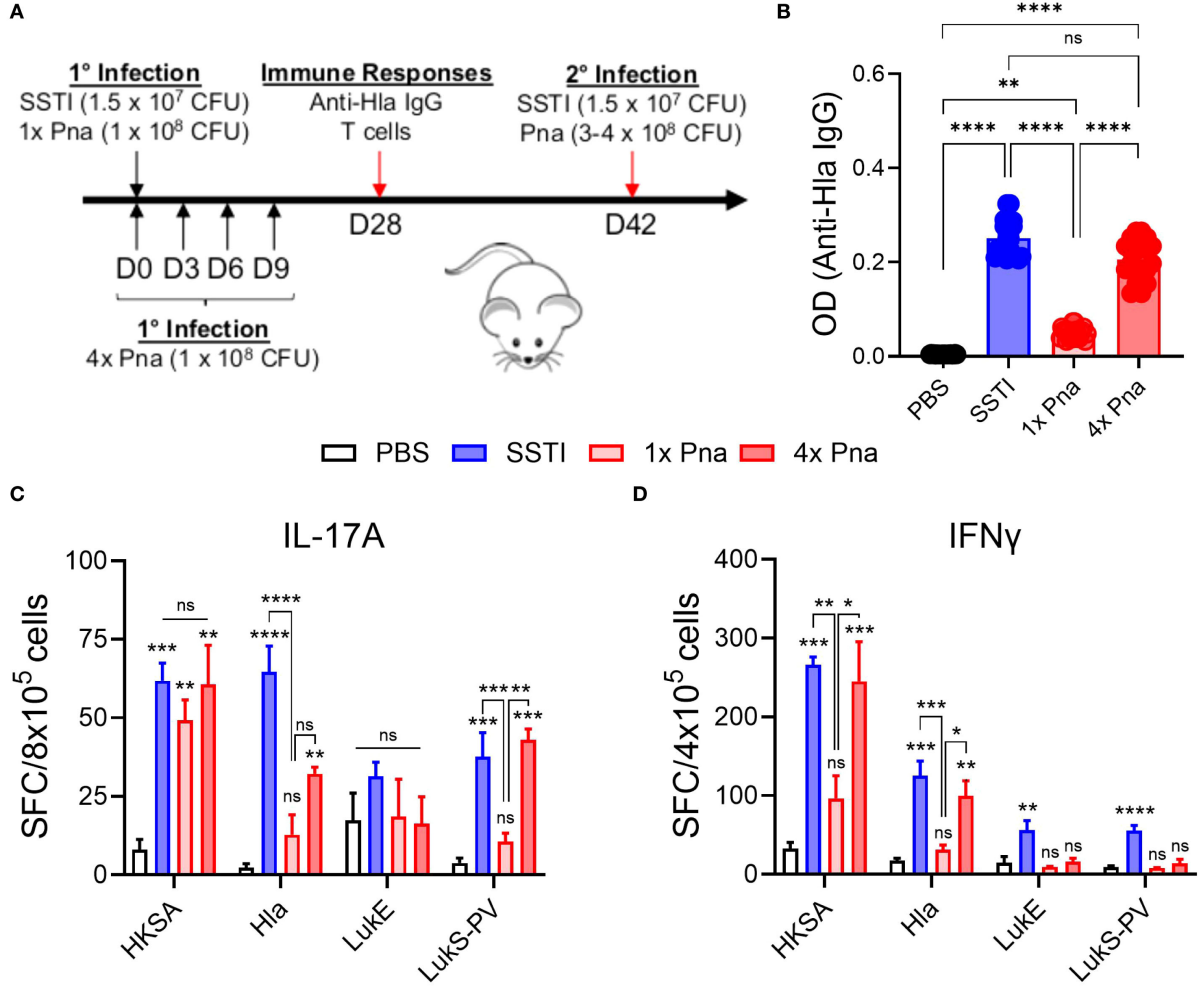
Immune responses following SSTI and transient and persistent pneumonia. **(A)** Mouse models of SSTI and pneumonia. Primary infection was induced by PBS, SSTI, transient pneumonia (1x pna), or persistent pneumonia (4x pna). For 4x pna, mice received repeated doses of S. aureus at 3 day intervals. Following primary infection, immune responses and protection against secondary infection were assessed (see **Figure 4**). **(B)** Anti-Hla IgG levels 28 days after infection n=20 mice/group. **(C, D)** Quantification of IL-17A- **(C)** and IFNγ- **(D)** secreting cells by ELISpot following culture of splenocytes with heat-killed S. aureus (HKSA) or purified toxins. N=5 mice/group from one representative experiment (of 2). All data are presented as mean ± SEM. Data were compared using one-way ANOVA with Tukey’s post-test. **(C, D)** Symbols above bars represent comparison with PBS group. *indicates p<0.05, **p<0.01, ***p<0.001, ****p<0.0001, ns indicates non-significant. SFC, spot-forming colonies.

Next, we compared immune responses following SSTI and transient (1x) or persistent (4x) pneumonia. Four weeks after primary infection, mice were euthanized, following which serum was collected for ELISA and spleens were collected for quantification of T cell responses (model, **Figure 2A**, see below for T cell responses). Consistent with our prior report (5), SSTI elicited high levels of Hla-specific IgG, but transient (1x) pneumonia did not (**Figure 2B**). In contrast, persistent (4x) pneumonia elicited high levels of Hla-specific IgG, comparable to SSTI. Therefore, bacterial persistence in the lung was sufficient to elicit Hla-specific antibodies.

### *S. aureus* persistence in the lung elicits T cell responses

In the same mice, we also quantified IL-17A and IFNγ-secreting T cells by ELISpot following culture of splenocytes with HKSA or purified Hla, LukE, or LukS-PV (model, **Figure 2A**). As we observed with antibody responses, there were more *S. aureus*- (HKSA), Hla-, and LukS-PV-reactive IL-17A secreting T cells following SSTI or 4x pna, compared with PBS, although the Hla-reactive IL-17A^+^ cells remained fewer than following SSTI (**Figure 2C**). Similarly, 4x pna elicited more Hla- and LukS-PV-reactive IL-17A^+^ cells compared with 1x pna. In contrast, there were no significant differences in LukE-reactive IL-17A^+^ cells among the groups (**Figure 2C**). For IFNγ, there were more cytokine-secreting T cells reactive against HKSA and Hla following SSTI or 4x pna, compared with PBS or 1x pna (**Figure 2D**). In contrast, only SSTI, but not 4x pna, elicited more LukE- and LukS-PV-reactive IFNγ-secreting T cells, compared with PBS or 1x pna (**Figure 2D**).

**Figure 3:**
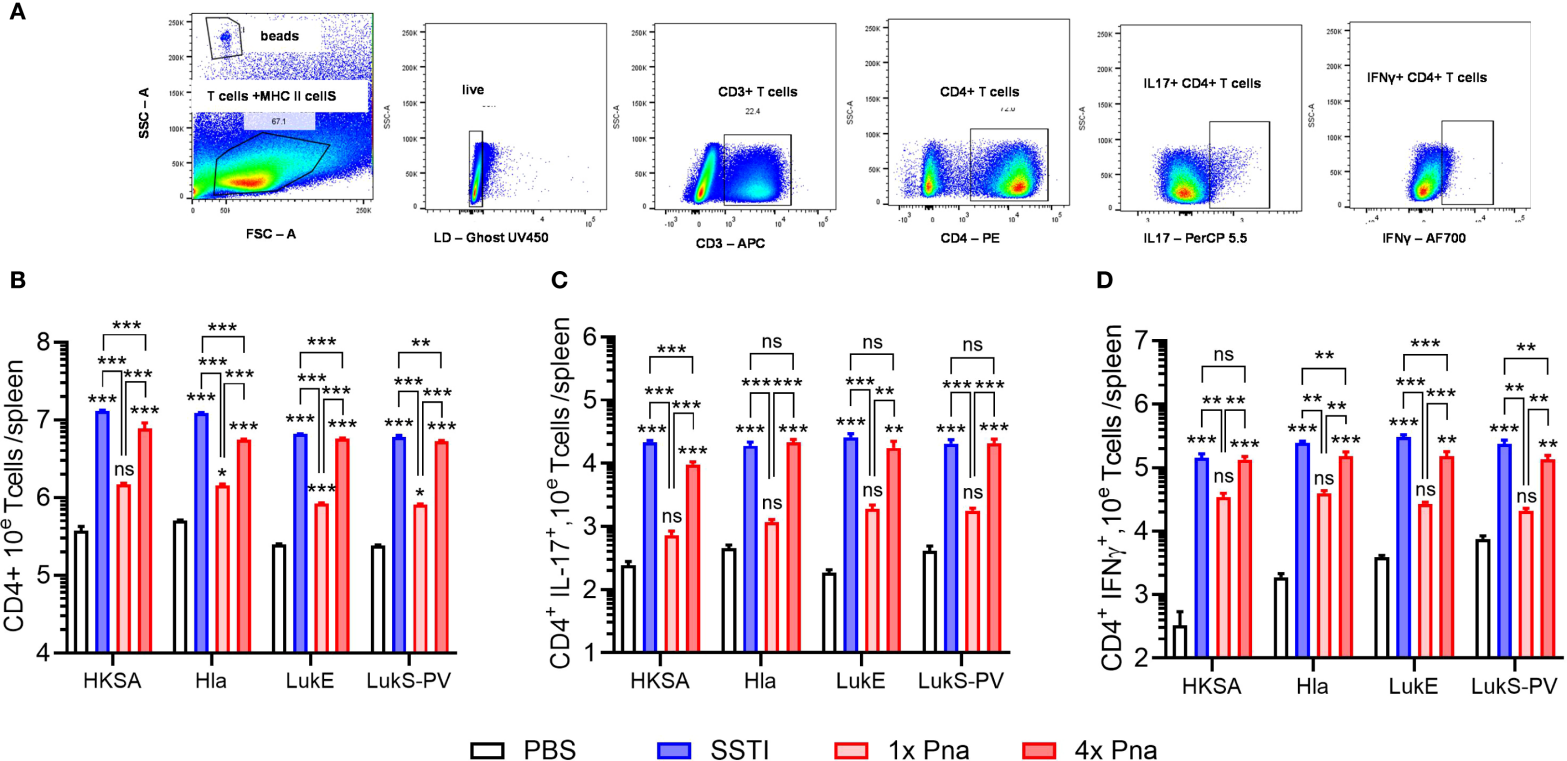
Expansion of CD4^+^ T cells following SSTI and pneumonia. **(A)** Gating strategy to quantify splenic CD4^+^ T cells. Mouse models are depicted in **Figure 2A**. CD4^+^ T cells were quantified in splenocytes of previously infected mice following culture with heat-killed S. aureus (HKSA), Hla, LukE, or LukS-PV. **(B)** Splenic antigen-specific CD4^+^ T cells. **(C)** CD4^+^ IL-17A^+^ antigen-specific T cells. **(D)** CD4^+^ IFNγ^+^ T antigen-specific T cells. SSTI – skin infection; 1x pna – transient pneumonia; 4x pna – persistent pneumonia. N=5 mice/group from one representative experiment (of 2), all data are presented as mean ± SEM. Data were normalized to total splenocytes and compared using one-way ANOVA with Tukey’s post-test. Symbols above bars represent comparison with PBS group.*indicates p<0.05, **p<0.01, ***p<0.001, ***p<0.001, ns indicates non-significant.

These results suggested that persistent pneumonia more strongly elicits expansion of antigen-specific T cells, but the ELISpot assay can neither quantify T cells nor identify specific T cell subsets. Therefore, we quantified CD4^+^ T cells by flow cytometry following culture of splenocytes with HKSA or the purified antigens following SSTI, 1x pna, or 4x pna (gating strategy, **Figure 3A**). Because the spleens of mice infected with SSTI or persistent pneumonia were considerably larger than those infected with pneumonia, indicative of a stronger systemic immune response, we corrected T cell numbers to account for the total numbers of T cells in the spleen. Consistent with enhanced expansion of T cells, there were more antigen-reactive CD4^+^ T cells (*S. aureus*- and toxin-specific) following 4x pna, compared with 1x pna, but the numbers remained modestly lower than those elicited by SSTI (**Figure 3B**). In contrast, while 1x pna elicited more toxin-reactive CD4^+^ T cells than PBS, there were markedly fewer CD4^+^ T cells compared with SSTI or 4x pna (**Figure 3B**). Similarly, while 1x pna resulted in a trend toward more CD4^+^ IL-17^+^ and IFNγ^+^ T cells than PBS, expansion of these cells was much stronger following SSTI and 4x pna (**Figures 3C, D**). As we observed with total CD4^+^ T cells, there were more *S. aureus*- (HKSA) reactive IL-17A^+^ T cells and toxin-reactive IFNγ^+^ T cells following SSTI, compared with 4x pna. However, there were no significant differences in toxin-reactive IL-17A^+^ T cells or *S. aureus*-reactive IFNγ^+^ T cells following SSTI or 4x pna. Taken together, these results demonstrate that bacterial persistence in the lung is necessary to elicit T cell expansion and functional cytokine responses.

### *S. aureus* persistence in the lung elicits protection against secondary SSTI and pneumonia

**Figure 4:**
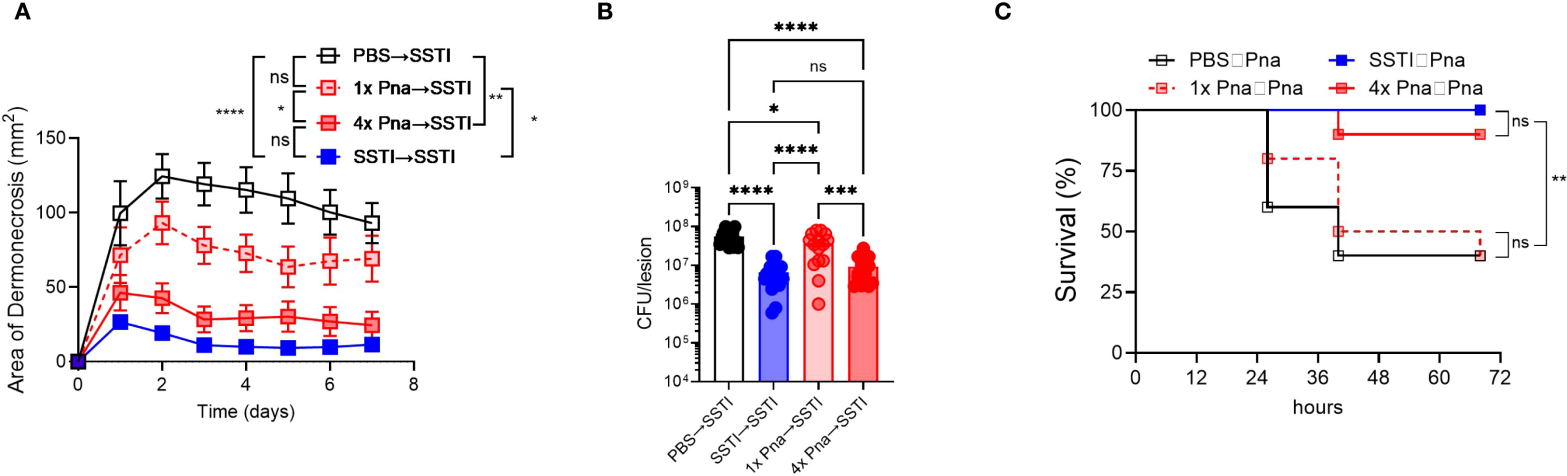
Persistent pneumonia protects against secondary SSTI and pneumonia. Mouse models are depicted in **Figure 2A**. **(A)** Protection against secondary SSTI following primary SSTI or pneumonia. SSTI – skin infection; 1x pna – transient pneumonia; 4x pna – persistent pneumonia. **(B)** Bacterial burden in the skin lesions 7 days after SSTI. **(C)** Protection against secondary pneumonia following primary SSTI or pneumonia. SSTI – skin infection; 1x pna – transient pneumonia; 4x pna – persistent pneumonia. **(A, B)** N=15 mice/group from 3 pooled experiments **(C)** N=10 mice/group pooled from two experiments. Data were analyzed by two-way ANOVA with repeated measures **(A)**, one-way ANOVA with Tukey’s post-test **(B)**, or Gehan-Breslow-Wilcoxon test **(C)**. All data are presented as mean ± SEM. *indicates p<0.05, **p<0.01, ****p<0.0001, ns indicates non-significant.

Based on the strongly elicited antibody and T cell responses following persistent pneumonia, we hypothesized that 4x pna would protect against secondary SSTI and pneumonia (**Figure 2A**, model). Indeed, dermonecrotic lesions during secondary SSTI in mice following primary SSTI or 4x pna were significantly smaller than naïve mice but not following 1x pna (**Figure 4A**). Similarly, there were fewer bacteria in the skin lesions following secondary SSTI in mice that received primary SSTI or 4x pna, compared with naïve mice, but not following 1x pna (**Figure 4B**). Consistent with stronger protective T cell and antibody responses, SSTI and 4x pna protected against secondary lethal pneumonia, but 1x pna did not (**Figure 4C**). Taken together, these results demonstrate that the stronger antibody and T cell responses elicited by bacterial persistence in the lung were accompanied by protection against secondary SSTI and pneumonia.

### Kinetics of immunologic memory development

**Figure 5:**
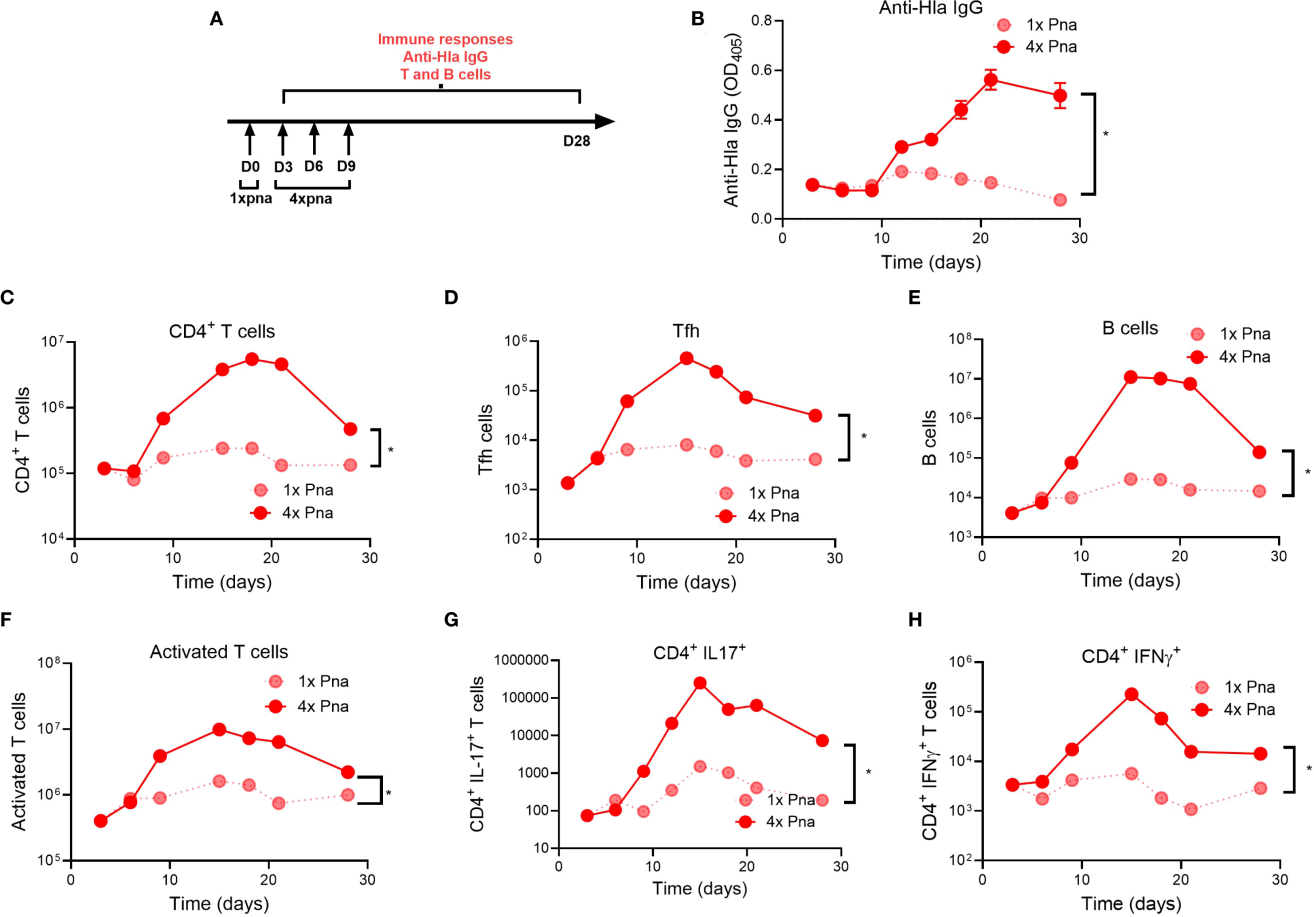
Kinetic development of adaptive immune responses following transient or persistent pneumonia. **(A)** Experimental models of transient (1x) and persistent (4x) pneumonia. **(B)** Anti-Hla IgG levels n=10. **(C–H)** *S. aureus*-reactive T and B cell responses were quantified following culture of splenocytes with heat-killed *S. aureus*. **(C)** CD4^+^ T helper cells; **(D)** follicular T helper cells (Tfh, CD4^+^ CXCR5^+^) **(E)** B cells (CD3^-^ CD19^+^); **(F)** activated T cells (CD44^+^); **(G)** IL17^+^ cells (Th17 cells); **(H)** IFNγ^+^ cells (TH1 cells). N=5 mice/group; for **(B)** results are presented as mean ± SEM; for **(C–H)** splenocytes of each group were pooled and normalized per spleen *p<0.1.

To better understand the kinetics under which antibody and T cell responses were driven by bacterial persistence, we quantified each following transient (1x pna) or persistent (4x pna) pneumonia (model, **Figure 5A**). For Hla-specific IgG levels, we found no significant differences early (3–9 days) after infection between 1x pna and 4x pna, but anti-Hla IgG levels increased significantly following 4x pna by day 12 and plateaued by day 28 (**Figure 5B**). These findings are consistent with our earlier demonstration of higher anti-Hla levels by 28 days following 4x pna (**Figure 2B**).

Next, we quantified lymphocyte populations that drive memory, including CD4^+^ T cells, follicular helper T cells (Tfh) that enable B cell and antibody development, and B cells. For all 3 cell populations, while there were no differences early after infection (<6 days) between 1x pna and 4x pna, there was greater expansion by 9 days, peaking between 15–20 days, after infection following 4x pna (**Figures 5C–E**), consistent with the later generation of Hla-specific IgG in these mice. Interestingly, each population modestly contracted by 28 days after infection. We also assessed effector T cell function by quantification of activated CD44^+^ T cells and IL-17A^+^ and IFNγ^+^ CD4^+^ T cells (representing Th17 and Th1 T cells, respectively). As we observed with memory T cells, activated and cytokine^+^ T cells increased by 9 days after infection following 4x pna, compared with minimal expansion following 1x pna (**Figures 5F–H**). Taken together, despite no differences early after infection, 4x pna resulted in expansion of B cells/antibody and memory and effector T cells as early as 9 days after infection, consistent with our findings of strong differences 28 days after infection (**Figures 2**, **3**).

## Discussion

To differentiate the impact of the site of infection and kinetics of bacterial clearance from infected tissues on elicited protective immunity, we developed a murine model to use as a tool to study the importance of bacterial persistence in eliciting immunological memory. We demonstrated that, in contrast to transient pneumonia with early bacterial clearance (“1x pna”), which failed to elicit strong antibody and T cell responses, persistent pneumonia (“4x pna”) strongly elicited *S. aureus*-specific antibodies and CD4^+^ T cells. These memory responses were similar in magnitude to those we previously reported following SSTI (5). The magnitude of adaptive immune responses was reflected in protection against secondary SSTI and pneumonia. These findings demonstrate that bacterial persistence in infected tissues is a key determinant of elicited immunologic memory and protection against recurrent infection.

Immunologic memory is required for protection against recurrent *S. aureus* infection. However, rapid clearance of *S. aureus* by the innate immune system may preclude the development of adaptive immunity due to transient exposure to antigens that would otherwise generate a memory response. Thus, transient exposure to *S. aureus* in the lung fails to achieve the threshold to elicit memory T cells and antibodies, since it takes time to stimulate proliferation and differentiation of memory and effector T cells (12). Memory T cell development has been observed with maximal responses closer to 14 days (13). Our findings of T cell expansion within 10 days of infection are consistent with this timeline. Since we found that *S. aureus* is cleared from the lungs and bloodstream within 6–8 days following transient (1x) pneumonia, it is perhaps not surprising that this transient exposure failed to elicit protective antibodies and memory T cells. It is also likely that there is an antigenic threshold necessary to elicit memory responses, such that the decreased numbers of bacteria recovered from the lung and blood on day 6 were insufficient to promote memory T cell expansion even though bacteria were still recovered. The necessity for bacterial persistence is not limited to the lungs, because we reported that antibiotic treatment of SSTI resulted in rapid bacterial clearance from the skin and failure to generate immunologic memory (14). Similar findings have also been reported for other pathogens. For example, antibiotic-induced clearance of *Chlamydia trachomitis* during genital infection inhibited development of protective immunity (8). Similarly, sustained persistence of *Salmonella typhimurium* and *Listeria monocytogenes* is necessary for generation of memory T cells and protective immunity (7, 15). Mechanistically, this is consistent with a study that used a mouse line engineered to express an MHC class II restricted epitope in dendritic cells to demonstrate that durable antigen persistence is essential for the CD4^+^ T cell expansion, even in the presence of an inflammatory stimulus (16). Taken together, these findings demonstrate that bacterial persistence is a fundamental driver of immunologic memory against pathogens.

Our findings of variability in T cell responses across the pneumonia and SSTI models may reflect more than just differences in antigen persistence. For example, we did not directly quantify antigen dose in our model; future studies are needed to discriminate between antigen dose and persistence. Factors such as epitope presentation, MHC binding affinity, antigen processing efficiency, inherent immunogenicity, the inflammatory milieu during priming, and frequency or duration of antigen exposure all play important roles in shaping T cell functionality.

In addition to these variables, the site of infection is also important in eliciting protective immune responses (17). In this study, we found that, despite comparably eliciting Hla-specific antibodies, SSTI elicited stronger T cell responses in the spleen compared with 4x pna. This correlated with stronger protection against SSTI. Thus, despite protection elicited by both SSTI and 4x pna, it is likely that SSTI elicited even stronger systemic immune responses. These results suggest that the site of infection can drive fundamentally different local and systemic immunity. Conversely, in humans, recurrent infection is less frequent following invasive infections, compared with non-invasive infections such as SSTI (4). This may be due to stronger induction of Hla-specific antibodies and antigen-specific memory cells following invasive infection (3, 17). In further support of the importance of the site of infection in elicited immune responses, transcriptomic analysis of 99 children with *S. aureus*-infection revealed that pneumonia was associated with stronger down-regulation of transcripts associated with B and T cell activation, compared with noninvasive infections such as SSTI (18). Therefore, the site of infection is an important determinant of elicited immune responses, but more work is needed to reconcile findings in mice and humans.

In mouse models of *S. aureus* infection, antibodies and T cells each contribute to protection (19–21). Our results are consistent with this notion, because persistent pneumonia (4x pna) elicited both Hla-specific antibodies and IL-17 and IFNγ-secreting T cells. Correlating with protection against secondary infection, anti-Hla IgG levels were highest following SSTI and 4x pna, but lower in 1x pna. Similarly, while 1x pna elicited some expansion of CD4^+^ IL-17^+^ and IFNγ^+^ T cells, compared with PBS, T cell expansion was weaker than that elicited by SSTI or 4x pna. These results are consistent with mouse and human studies that demonstrate the importance of CD4^+^ Th17 and Th1 cells in protection (22, 23). Importantly, Hla-specific antibody and T cells synergize to protect against *S. aureus* (24, 25). While both antibodies and T cells contribute to protection against secondary SSTI and pneumonia, future studies will identify specific roles for antibodies and T cell subsets, including circulating and tissue-resident memory (T_RM_) T cells, in protection elicited by persistent pneumonia.

There are several limitations to the study, First, the mouse models may not accurately recapitulate human infection. For example, our model of “persistent” pneumonia, in which mice are repeatedly dosed with a low inoculum of *S. aureus*, does not reflect the reality of bacterial persistence in humans. Rather, we leveraged this model as a tool to study bacterial persistence because bacteria are otherwise rapidly cleared from the murine nasopharanyx and lung. Moreover, we observed strong generation of protective antibody and T cells following SSTI and persistent pneumonia in mice, but in humans the strongest immune responses are observed following invasive infections, compared with noninvasive infections such as SSTI (4, 19). Second, we did not determine if protection elicited by persistent pneumonia is mediated by antibodies, T cells, or a combination thereof. Future studies will address the specific roles of antibodies and T cells in protection. Third, the cytokine-secreting cells detected by ELISpot may not solely be T cells, because NK cells and other innate lymphoid cells also produces IL-17A and IFNγ (26, 27). However, our flow cytometry experiments confirm that T cells are a major source of cytokine production. Similarly, the current study indicates that CD4^+^ T cells are the primary source of IL-17 among circulating T cells. Nonetheless, we cannot rule out the possibility that γδ T cells are a major source of IL-17 in lung and skin tissues. Fourth, we elected to quantify T cells in the spleen as a measure of systemic immunity. Future studies will interrogate local immune responses in the lung (e.g. lung T_RM_) and draining lymph nodes.

Finally, our current study does not directly address the influence of pre-existing or immediate immune microenvironments on the relationship between antigen exposure duration and memory responses. We note that the mice used in our experiments were naïve to *S. aureus*, as confirmed by the absence of detectable antibody responses in PBS-treated controls. However, a major focus of ongoing work in our group is to understand how prior *S. aureus* exposure shapes both infection- and vaccine-elicited immune responses. Additionally, it is highly plausible that the immediate immune microenvironment, particularly tissue-specific factors in the lung versus skin, plays a critical role in directing immune responses. While beyond the scope of the current study, this is an important area for future investigation.

In summary, our findings demonstrate that bacterial persistence is essential for mature antibody and T cells responses and, consequently, for protection against secondary infection. These findings highlight the importance of both site of infection and bacterial persistence in infected tissues in eliciting protective immunologic memory.

## Data Availability

The raw data supporting the conclusions of this article will be made available by the authors, without undue reservation.
